# Reply to: Need for improvements in reported cost-effectiveness of adalimumab in rheumatoid arthritis

**DOI:** 10.1007/s00393-016-0256-2

**Published:** 2017-01-13

**Authors:** C. Gissel, G. Götz, H. Repp

**Affiliations:** 1 Junior Chair of Health Economics, Justus Liebig University Giessen, Licher Str. 62, Giessen, 35394 Germany; 2 Chair for Industrial Organization, Regulation and Antitrust, Justus Liebig University Giessen, Giessen, Germany; 3 Department of Medicine, Justus Liebig University Giessen, Giessen, Germany

## Reply

This reply refers to Weber S, Pongratz G, Schneider M, Brinks R Need for improvements in reported cost-effectiveness of adalimumab in rheumatoid arthritis. doi:10.1007/s00393-016-0255-3

Original version: Gissel, Götz, Repp (2016) Cost-effectiveness of adalimumab for rheumatoid arthritis in Germany. doi:10.1007/s00393-016-0071-9

We thank Sergej Weber, Georg Pongratz, Matthias Schneider and Ralph Brinks for acknowledging the relevance of health economic modeling for biologic treatments for rheumatoid arthritis. We take this opportunity to comment on some health economic concepts that our model is based on:

1. Concerning transition probabilities: We set up an individual patient sampling model that simulates each patient individually based on the treatment algorithms and the specific treatment duration parameters as specified or cited in the paper’s “Model and methods” section. The model does not rely on fixed transition probabilities.
2. Concerning switching between the two study arms (adalimumab arm, control arm): The purpose of having a control arm is the ability to attribute clinical and economic effects to the introduction of adalimumab with the smallest possible bias. Switching is impossible by design.
3. Concerning Weber and colleagues’ request for further discussion of various aspects in their 7500+ character letter: The journal deserves our gratitude for taking into consideration our 30,000+ character manuscript despite its 25,000 character limit. We honored the journal’s brevity requirements as much as possible and we stuck to citations whenever possible.
4. Concerning quality of life: Contrary to Weber and colleagues’ suggestion, the *absolute* levels of quality of life, which they estimated from [1], cannot be compared to the *relative* gains of quality of life in our model.
